# Presenting segmented images in a rapid serial visual presentation stream improves search accuracy

**DOI:** 10.1186/s41235-025-00653-2

**Published:** 2025-08-15

**Authors:** Krystina Diaz, Mark W. Becker, Chad Peltier, Jeffrey B. Bolkhovsky

**Affiliations:** 1 Leidos Inc., Reston, VA USA; 2 Michigan State University (MSU), East Lansing, MI USA; 3 Naval Submarine Medical Research Laboratory (NSMRL), Groton, CT USA

**Keywords:** Rapid serial visual presentation, Visual search, Eye movements, Attention

## Abstract

Visual search performance is a critical factor in many high-stakes duties, warranting the need for strategies to enhance target detection accuracy. Research using rapid serial visual presentation (RSVP) of stimuli shows that observers can detect categorically defined, pre-specified targets even when the presentation rate is rapid, suggesting RSVP as a viable strategy. To investigate how and how well RSVP can improve target detection in complex search arrays, five experiments were conducted to compare search performance between Full-Image search conditions and various RSVP-based conditions. Stimulus presentation time/total search time was the same across conditions. Experiment 1 demonstrated the utility of RSVP to enhance target identification in simple arrays (i.e., Landolt Cs). Experiment 2 involved more complex scenes and target-present/-absent judgments. Results showed that RSVP increased target detections due to both a liberal change in criterion and an increase in sensitivity. Experiment 3 provides some evidence against the reduction in peripheral clutter as the primary contributor to RSVP performance increases. Experiments 4 and 5 prompted and limited eye movements, respectively, to distinguish the role of eye movements in RSVP-based search. These two latter experiments imply that lower target detection performance under time constraints in whole image search conditions is attributable to time-wasting, irrelevant and inefficient eye movements. These experiments suggest that RSVP advantage occurs because the method maximizes time for inspecting and processing each search image/segment. Real-world visual search tasks may benefit from segmenting the search display and presenting images in an RSVP stream.

## Introduction

Many real-world search tasks require an observer to search an environment (e.g., an image, a display, a crowd of people) and determine whether a critical target is present. For example, a radiologist decides from a magnetic resonance imaging (MRI) scan whether or not a tumor is present, a transportation security administration (TSA) baggage screener determines from a luggage scan if a bag contains contraband (e.g., a weapon), and a military unmanned underwater vehicle (UUV) operator identifies the presence of a foreign object (e.g., a mine on the sea floor) on a UUV control display. Though the goal of these crucial searches is to find a target item when a target is present, even trained experts still miss targets (Krupinski, 1996; Nodine & Kundel, 1987; Nodine et al., 1996; Schwaninger et al., 2005) which may lead to severe consequences.

Many types of misses may result from observers failing to inspect the region of an environment that contains the target (Godwin et al., 2015b). For example, it has been estimated that 24%−36% of missed targets in mammography can be attributed to radiologists failing to search the area of the mammogram containing the lesion (Bird et al., 1992; Krupinski, 1996), with radiologists inspecting only approximately half of an image before making a “target-absent” decision and concluding that no lesion is present (Krupinski, 1996). Research has also suggested that rapid and incomplete searches by TSA agents may result in failure to inspect the critical region of a bag containing contraband, thereby leading to missed threats (Wolfe et al., 2013). Laboratory-based studies have found that regardless of search difficulty, failing to visually fixate on a target or quitting search before inspecting the entire search area are the most common visual search errors—called “selection errors” (Fleck & Mitroff, 2007; Ishibashi et al., 2012; Teo et al., 2013; Wolfe et al., 2005; Zenger & Fahle, 1997).

Additional types of errors in which observers fixate on a target but still make a target-absent determination ("identification errors”) have been attributed to shifts in observer decision bias. With these types of errors, the likelihood of observers making a target-present or target-absent decision fluctuates between being more liberal (higher likelihood of making a target-present determination) or more conservative (higher likelihood of making a target-absent determination) (Wolfe & Van Wert, 2010). Identification errors, the failure to recognize a target when fixated, are dependent on search factors such as target prevalence or temporal proximity to a previous target detection (Becker et al., 2020; Green & Swets, 1966; Peltier & Becker, 2016; Wolfe & Van Wert, 2010). Whereas selection errors account for 75–85% of target misses, identification errors account for 15–25% of target misses (Peltier & Becker, 2016).

The relative contribution of selection vs. identification error implies that to improve target detection performance, one should implement visual search techniques capable of 1) maximizing the total search area that observers inspect, and 2) minimizing the potential for quitting search early or shifting decision criteria. Strategies for enhancing performance include allowing observers more time to search or preventing observers from manually terminating search early (Wolfe et al., 2007). However, these strategies may not be practical, as real-world demands put pressure on an observer to perform their job quickly. For example, a radiologist cannot neglect other patients to focus extensively on one patient’s scan, and a UUV operator must detect the presence of a mine before it detonates. Moreover, there is no guarantee that looking at an image longer means that observers are actively searching it longer; they may reach an internal quitting threshold and stop actively searching regardless of whether they have made a target-absent response. Given real-world temporal constraints on visual search, an ideal method of improving search would instead allow an observer to inspect a greater amount of the search environment without increasing search time.

One way of increasing the total area of an image searched within a fixed amount of time is to present the search scene as small segments, in a rapid serial visual presentation (RSVP) stream, at fixation. The entire scene is presented in digestible pieces, and because RSVP presents each segment automatically and controls the presentation time of each segment, observers are unable to terminate search prematurely (i.e., cannot manually terminate a search trial early), reducing the opportunity for selection errors. Experiments have shown that when a participant knows the identity of a target beforehand, they can detect the target in an RSVP stream in which each image is presented very briefly (Intraub, 1981; Potter et al., 2002). For instance, when the RSVP image segments were relatively small and presented at fixation, participants could identify a target’s presence, significantly above chance, even when the segments were presented for only 13 ms each (Potter et al., 2014). The speed with which observers can identify targets within an RSVP stream supports RSVP’s potential to increase search efficiency for visual search tasks in which overall search time is limited. Thus, compared to “Full-Image” visual search in which a search array is displayed in its entirety, RSVP yields enhanced target identification (Potter et al., 2014).

To investigate this, Forlines and Balakrishnan (2009) compared visual search performance in which overall search time remained constant, and the search array stimulus was presented either in a full image or RSVP stream segments. The search array consisted of a series of letters, and participants made a target-present response when a vowel was present. The researchers found higher detection accuracy in the RSVP condition, suggesting that this method of stimulus presentation may be effective in reducing missed targets. However, the stimuli utilized in Forlines and Balakrishnan (2009) (i.e., letters of the English alphabet) could be considered highly familiar stimuli, making target detection easy for participants. Additionally, the search arrays/stimuli were presented in a very simplified manner, in which a single letter would comprise a single segment, thereby minimizing the visual search effort required for target detection and simplifying the task toward one of recognition as opposed to search (Kotowicz et al., 2010). The generalizability of the effects of RSVP using more complex arrays warrants additional investigation.

The present study continues the exploration into whether the RSVP method of presenting a search display improves target detection, and if so, by what mechanism(s). Knowing such mechanism(s) of improvement can facilitate the implementation of similar, RSVP-like stimulus presentation methods into real-world visual search environments (e.g., using RSVP to present luggage scans to TSA agents). The following series of experiments compared target detection performance in Full-Image control conditions to that of RSVP conditions that varied in presentation method and complexity. We hypothesized that RSVP conditions would yield enhanced target detection accuracy. Full-Image control conditions presented each search array as a whole image, for fixed periods of time. The RSVP conditions presented equally sized segments of each stimulus image, in RSVP streams at or near fixation. Each segment was presented briefly and for the same amount of time such that the overall search times in the RSVP conditions were equated to the search times in the Full-Image conditions. Participants in each stimulus presentation condition were required to detect pre-specified targets from the stimuli images.

Experiment 1 served as a conceptual replication of Forlines and Balakrishnan (2009). Similar to that study, simple arrays were used as stimuli, in which only a single stimulus/letter was presented per segment, though the stimuli presented were less familiar characters (Landolt Cs), the location of the letter in each segment appeared near (but not directly at) fixation due to random jitter applied during RSVP segmentation, and the RSVP segment presentation times were reduced (150 ms compared to 200 ms). In Experiment 2, the RSVP method was extended to more complex images and stimulus arrays (“Where’s Waldo” scenes; Handford, 2007). Experiment 3 tested if RSVP’s benefit could be explained by differing levels of peripheral clutter, a factor which has been linked to search performance (Beck et al., 2010; Henderson et al., 2009; Rosenholtz et al., 2007). Experiment 4 investigated the role of eye movements in RSVP-based search, and attempted to guide search and therefore control eye movements through the presentation of RSVP segments across a display screen. Lastly, Experiment 5 built upon Experiment 4 to reduce the overall non-search-related eye movements via RSVP segment cueing, and assessed subsequent impacts on search performance. These experiments test the extent of RSVP performance effects and explore the mechanisms behind RSVP-based visual search performance.

## General methods

All participants across all experiments were undergraduate students, aged 18 or older, from Michigan State University (MSU). All participants had normal or corrected-to-normal vision (including normal color vision) and participated in one of the five experiments to fulfill course credit. Each experiment recruited from a new, randomly selected participant pool. All experimental protocols were approved by Michigan State University’s Institutional Review Board.

To estimate sample sizes for the five experiments, we conducted a power analysis using G*Power (Faul et al., 2007). For those experiments involving paired samples t-tests (Experiments 1–3) seeking to detect a moderate effect size (d = 0.5) at alpha level α = 0.05 and power = 0.80, the minimum number of participants is 34 participants. For those experiments involving within-subjects one-way analyses of variance (ANOVAs) seeking to detect a moderate effect size (f = 0.25) at alpha level α = 0.05 and power = 0.80, the minimum number of participants is 34 participants for a three-condition (three visual search presentation conditions) study (Experiment 4) and 29 participants for a four-condition (four visual search presentation conditions) study (Experiment 5). Following Experiment 1, to account for participant attrition and/or data being excluded from final analysis due to below-chance performance (see “Results and Discussion” sections for more information), we attempted to recruit at minimum 20% more individuals than recommended for each experiment.

Stimuli presentation/search arrays for all experiments were programmed in E-prime (Psychology Software Tools, version 2.0; Schneider et al., 2002). Stimuli were presented on an 18.1-inch CRT monitor set at a resolution of 1024 × 768. Participants viewed the monitor from approximately 55 cm, and used buttons on a serial response box (Psychology Software Tools, Pittsburgh, PA) connected to the computer to indicate their target detection responses. All stimulus presentation/search conditions provided auditory feedback upon participants submitting an incorrect response. The order of which condition participants completed first was counterbalanced, in all experiments.

Based on a pilot study (N = 12) in which participants could self-terminate search trials (via button press) during a Full-Image search, 3600 ms was found to be a sufficient and adequately challenging (i.e., would not cause target detection performance ceiling effects) amount of search time per trial. On average, pilot participants searched for 4212 ms (*SEM* = 271.41) with a mean hit rate (correct target detections) of 88% *(SEM* = 3.60%). In all five of the current experiments, stimuli/trials were presented for less than this amount of time to create time pressure. The intent was also to present each image in the RSVP stream quickly enough to limit eye movements wandering away from the stimuli/to other areas of the screen prior to the next stimulus presentation. Total search time was divided by the number of segments created for each RSVP stream (segment numbers were dependent on scene/image sizes for each experiment) to come up with an equivalent amount of search time per RSVP segment.

## Experiment 1: RSVP in simple scenes

### Methods

#### Participants

Thirty-seven participants, in a within-subjects design, completed visual search tasks in a Full-Image condition, which consisted of a full array of search stimuli, and an RSVP condition.

#### Stimuli

Each search array consisted of 24 colored Landolt Cs against a black background. Each “C” was an outline of a circle that subtended 0.625 degrees of visual angle with a small 0.104-degree break. The line width of each C was 0.104 degrees. For the 23 distractor stimuli, the break randomly appeared either at the top, bottom, or right side of the “C.” The target “C” had the break on the left.

**Fig. 1:**
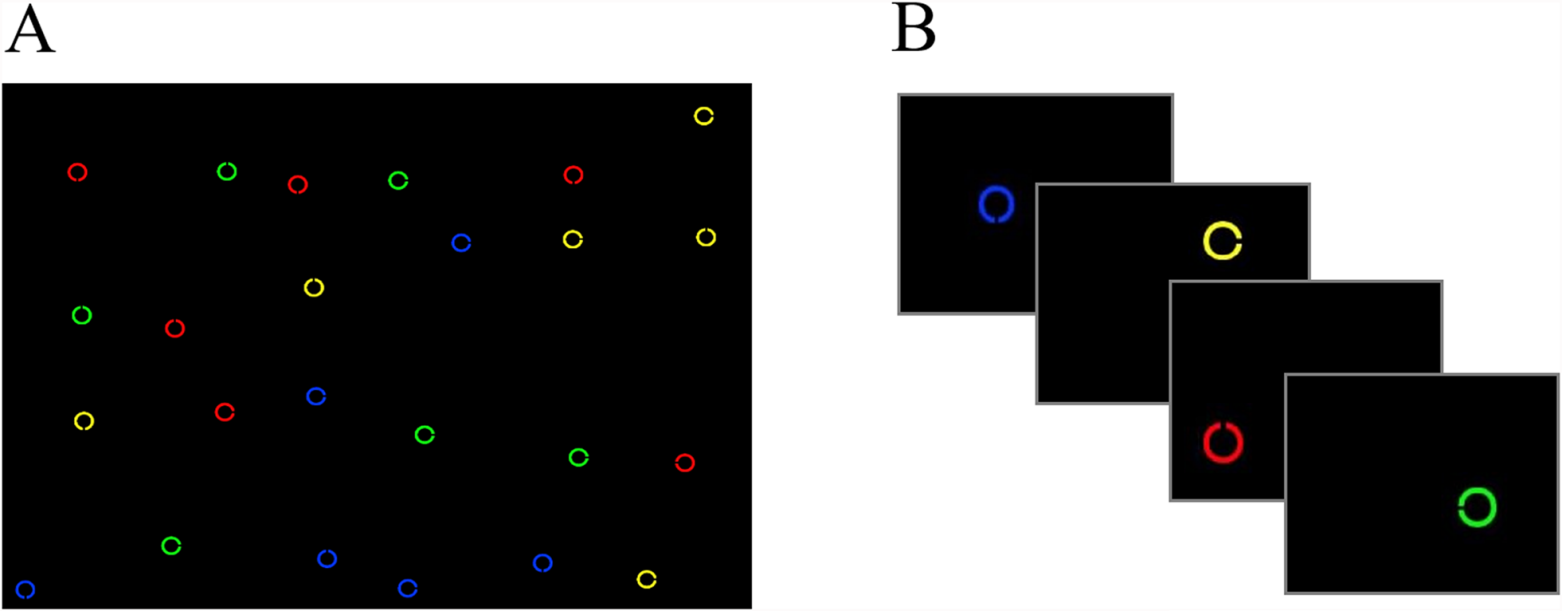
*Experiment 1 Search Stimuli.* (**A**) Full-Image condition in which stimuli were presented as complete, static images. (**B**) RSVP condition, in which stimuli (segmented full images) were presented sequentially. The stimuli shown here are enlarged, whereas during the experiment, each segment was 1/24^th^ the size of the Full-Image condition stimuli images. The fourth image depicts the target “C”

To create images (search arrays) for the Full-Image condition (see Fig. 1A), the screen area was divided into 24 (a 6 by 4 matrix) equal-sized (6.4° × 7.1°) regions. A single Landolt C was placed within each region, with random jitter that allowed the “C” to appear anywhere within the region. This jitter broke-up the orderly organization of the matrix and resulted in “C”s appearing in different locations across trials. The color of each “C” was randomly assigned to one of four highly discriminable colors (red, green, blue, and yellow), with the provision that each display consisted of six items of each color.

For the RSVP condition, streams of 24 images were created by sequentially presenting each of the 24 regions from a Full-Image display as segments, at fixation (see Fig. 1B). This resulted in a single “C” appearing in each segment of the RSVP stream. Given the random jitter of “C”s within each region, each “C” could appear anywhere within a 6.4° × 7.1° region centered at fixation. This jitter was implemented because placing each “C” directly at fixation would have allowed people to monitor a single location (the left side of the “C” at fixation) for the target. The jitter did not allow for such a strategy.

A target was present on each trial, and each of the four colors was equally likely to be a target. In the Full-Image condition, each image was considered a single experimental trial, whereas in the RSVP condition, each RSVP stream of 24 segments was considered a single trial. The target was equally likely to appear in any region of the Full-Image condition, and was equally likely to appear in any segment within the stream of the RSVP condition.

Although the broader motivation for this work is based in real-world search tasks, many of which involve determining whether a target is present or absent, Experiment 1 requires that participants identify the color of the object that contains a target. This choice reflects the design of previous work that Experiment 1 replicates and extends. Though the task format differs from later experiments (Experiments 2–5) that adopt a more externally valid target-present/-absent design, Experiment 1 nonetheless serves to validate key comparisons across display conditions under controlled conditions of known target presence.

#### Procedure

Participants were informed that their task was to search for and report the color of the left-facing target “C” among a number of right, upward, and downward-facing, non-target “C”s. In the Full-Image condition, a search array was presented for 3600 ms before the participant was prompted to make a key press to indicate the color of the target “C.” Trials could not be terminated with an early button press. In the RSVP condition, each image segment within a stream was presented for 150 ms and was immediately replaced by the next image in the stream, for a total search time of 3600 ms per trial (see Fig. 1B). Participants responded at the end of the stream and could not terminate the trial with an early button press. Participants completed one block of 80 trials for each condition. The total time of the search task, per condition, was approximately 4 minutes and 48 seconds. The order of the blocks (i.e., the order of which condition participants completed first) was randomized across participants.

## Results and discussion

**Fig. 2:**
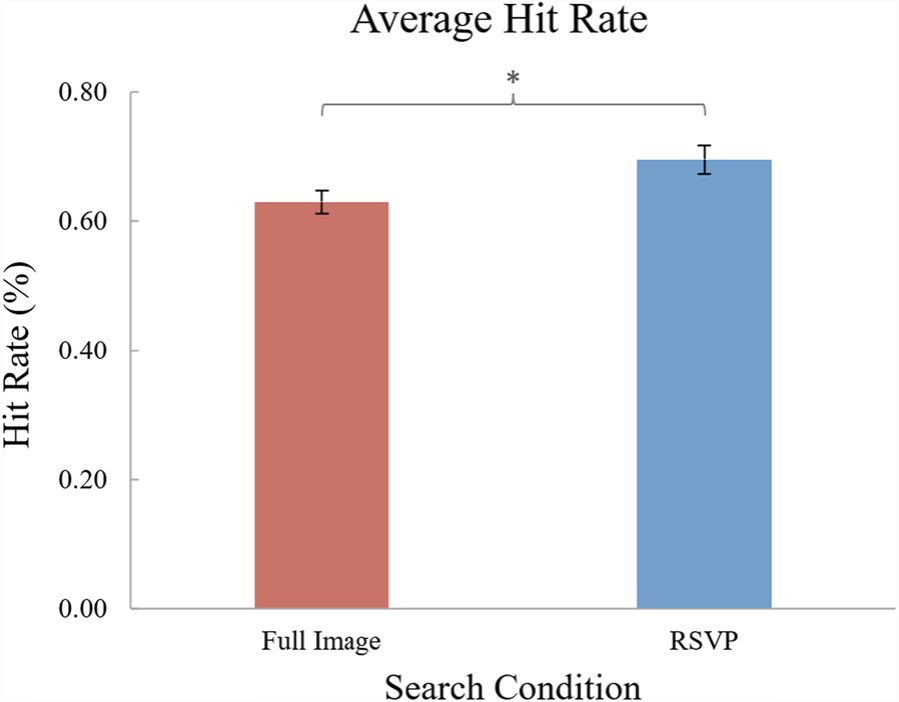
*Experiment 1 Results.* The hit rate for each search condition. Error bars represent the standard errors of the means. (*) represents a significant within-subjects comparison ($\alpha =0.05$)

Four participants were excluded from analysis for accuracy below chance (25%), leaving 33 participants’ data for analysis. The exclusion of the four participants’ data did not alter the interpretation of the data. A paired samples t-test found that average hit rate was significantly higher [($\alpha =0.05$), *t*(32) = 2.38, *p* = .024, *d* = 0.41 (Cohen’s *d*)] in the RSVP condition (*M* = 69.55%; *SEM* = 0.02) than in the Full-Image condition (*M* = 62.95%, *SEM* = 0.02) (see Fig. 2).

The relatively low target detection rates in the Full-Image condition suggest that 3600 ms of search time per trial produced time pressure; participants likely did not search the entire display in this amount of time, but still performed well enough to avoid floor effects. The RSVP method did appear to be less affected by this time pressure, suggesting that participants viewed every part/segment of the search array. Experiment 1’s results conceptually replicate Forlines and Balakrishnan (2009); observers were more accurate in identifying simple targets in RSVP streams than in Full-Image searches. In addition, the current experiment extends their work to show that the RSVP method provides a performance benefit in search environments that include less familiar stimuli that are presented at a faster rate and with spatial uncertainty (i.e., off-fixation).

These results, together with those of Forlines and Balakrishnan (2009), imply that the RSVP method is an effective method for improving search performance while holding the overall allotted search time constant. However, in both studies, the stimuli consisted of simple character/object arrays, in which each object was visually salient from the background. In addition, there was one object on the screen at a time during each RSVP stream, and so it is unclear how well the RSVP advantage would generalize to more complex scenes that better reflect the types of scenes viewed in real-world applications.

To address these issues, Experiment 2 investigated whether the RSVP method would show a similar performance benefit for search of complex (e.g., cluttered) scenes rather than simple arrays. Participants searched “Where’s Waldo” images for the presence or absence of the character Waldo.

## Experiment 2: RSVP in complex scenes

### Methods

#### Participants

Forty-five participants completed visual search tasks in a Full-Image condition (full “Where’s Waldo” images) and an RSVP condition (streams of equally sized “Where’s Waldo” image segments) in a within-subjects design.

#### Stimuli

Stimuli consisted of 18 unique “Where’s Waldo?” scenes. To create target-absent scenes, Adobe Photoshop (Adobe Systems, San Jose, CA, USA) was used to remove the original target (“Waldo”) from each scene. The area that had contained the target was filled in using other parts of the image. It is possible that this minor modification produced low-level visual discrepancies that could influence search behavior; however, this would have been the case in both the Full-Image and RSVP conditions, and therefore, any potential confounds were balanced across conditions. To create target-present scenes, one standard target (image of “Waldo”) was used throughout the experiment and edited into scenes; the original “Waldos” were edited out of their images. The target subtended 1° in width and 1.6° in height.

**Fig. 3:**
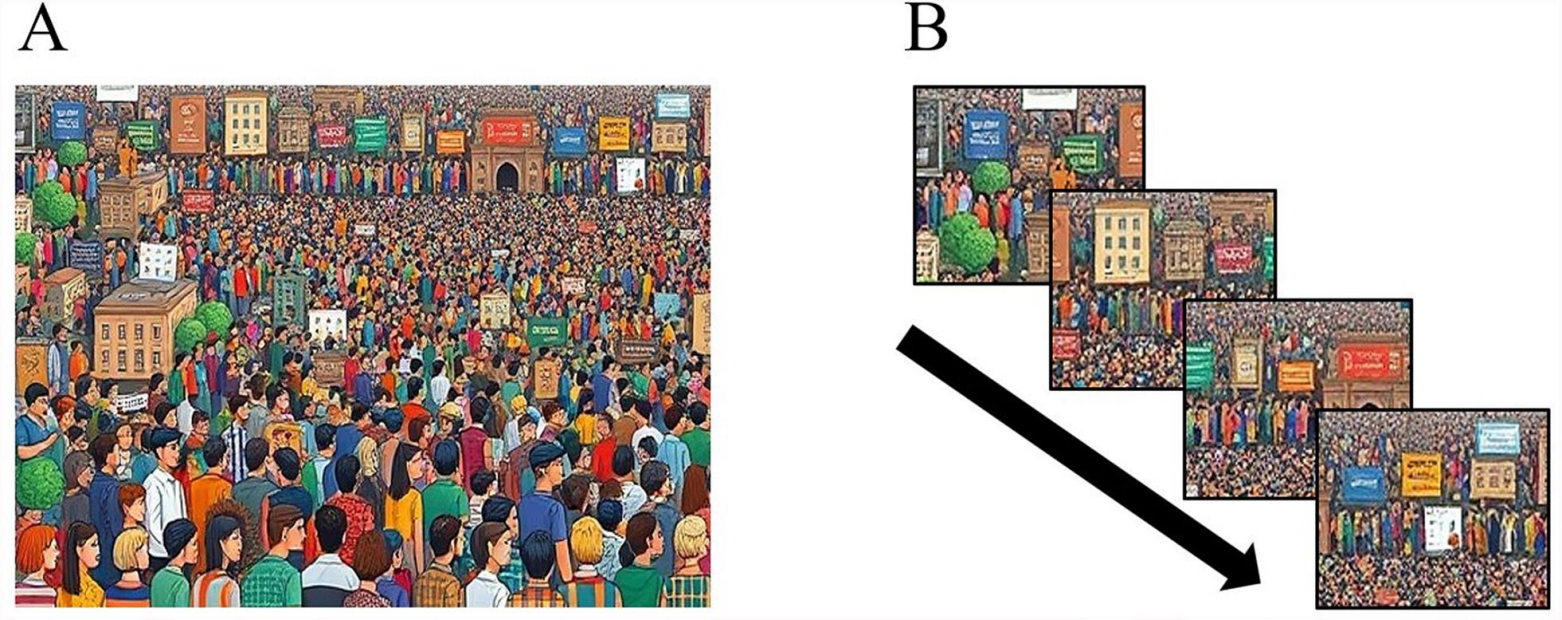
*Experiment 2 Search Stimuli.* (**A**) Full-Image condition, in which stimuli were presented as complete, static images. (**B**) RSVP condition, in which stimuli (segmented full images) were presented sequentially. The stimuli shown here are enlarged in the RSVP panels, whereas during the experiment, each segment was 1/16^th^ the size of the Full-Image condition stimuli images. [Please note that due to copyright, these are AI generated replicas of the original Where’s Waldo images, used for this article only]

The Full-Image condition presented an edited Where’s Waldo scene at the full size of the display screen (see Fig. 3A). A target would appear on 50% of the trials and would be placed in a random location. To create streams for the RSVP condition, the Waldo scenes were segmented into 16 equally sized (9.6° by 7.1°) regions. These segments were then presented sequentially in an RSVP stream (see Fig. 3B). The target was placed in a random location within one of the segments in 50% of the trials. To account for any serial position effects, the target could not appear in the first or last segment, but otherwise could appear in the other segments with equal probability. Each of the 16 segments was displayed for 150 ms at fixation. Note that compared to Experiment 1, there were fewer segments per stream, and that each segment was slightly larger. This was done to maintain the target area-to-segmented area ratio in the RSVP stream. Given the larger target, the RSVP streams required larger segments.

#### Procedure

Participants were shown an image of “Waldo” to familiarize them with the target. They were told to search for it among the display. In the Full-Image condition, each trial consisted of a randomly selected (with replacement) “Where’s Waldo” scene that was presented for 2400 ms. In the RSVP condition, each trial consisted of a 16-segment RSVP stream with each segment presented for 150 ms, for a total search time of 2400 ms. In both conditions, participants were prompted to make a target-present/absent judgment after the 2400 ms display period finished. Participants could not manually terminate a display period early (i.e., could not terminate a trial prematurely via button press); the search task would only progress to the next trial when participants responded to the prompt and made a target-present or target-absent determination. Participants completed one block of 100 trials for each condition. The search task in each condition lasted approximately 4 minutes. The order of the blocks was randomized across participants.

## Results and discussion

One participant’s data were excluded from analysis for total accuracy (hits + correct rejections) being below chance (50%), leaving 44 participants’ data for analysis. The exclusion of this participant’s data did not change the interpretation of the data. The investigated performance metrics included hit rate, false alarm rate (the percentage of incorrect target-present responses), and corrected hits (hit rate – false alarms)—a method of assessing the performance tradeoff between hit rate and false alarms and of gauging sensitivity of a search condition to accurate target identification.

A paired samples t-test found that participants’ average hit rate was significantly higher, *t*(43) = 7.97, *p* < .001, *d* = 1.20, in the RSVP condition (*M* = 65.15%; *SEM* = 0.02) than the Full-Image condition (*M* = 49.56%; S*EM* = 0.02). However, participants’ average false alarm rate was significantly higher, *t*(43) = 3.12, *p* = .003, *d* = 0*.*47, in the RSVP condition (*M* = 16.54%; *SEM* = 0.02) than the Full-Image condition (*M* = 10.49%; S*EM* = 0.02). The elevated false alarm rates (see Fig. 5A) could imply that the increased hit rate in the RSVP condition may have been created by a more liberal shift in decision criterion (Green & Swets, 1966). In other words, participants may have required increasingly less target-qualifying evidence accumulated from the search display during RSVP trials to make target-present responses. Therefore, participants may have become more likely to classify fixated items as targets (as opposed to non-targets). A paired samples t-test on corrected hits (hits – false alarms) found significantly higher performance, *t*(43) = 4.61, *p* < .001, *d* = 0.70, in the RSVP condition (*M* = 48.61%; *SEM* = 0.02) than the Full-Image condition (*M* = 39.07; S*EM* = 0.02), suggesting that RSVP presentation not only changed the criterion, but also increased sensitivity to target identification.

To confirm this change in sensitivity, each participants’ *d* (d-prime) statistic was calculated in which the *d* value represents sensitivity to target detection above chance (0). A paired samples t-test confirmed that sensitivity was significantly higher, *t*(43) = 2.56, *p* = .014, *d* = 0.39, in the RSVP condition (*M* = 1.40; *SEM* = 0.09), than the Full-Image condition (*M* = 1.05; *SEM* = 0.10).

These data revealed that the RSVP method significantly increased false alarms, implying a shift in decision bias toward a target-present response. However, this is accompanied by a greater increase in hits, as shown by the significantly higher corrected hits and *d* statistics in the RSVP condition. Thus, even though the false alarm rate increased, sensitivity when using RSVP during search was greater than in a Full-Image search. These results expand on Experiment 1 and Forlines and Balakrishnan (2009), showing that the RSVP method increases accuracy in complex search displays. Notably, this supports the viability of the RSVP method in complex search tasks, rather than only in tasks with simple stimuli.

The researchers hypothesized that the RSVP method could result in improved target detection even in these more complex scenes because it reduces the overall clutter that is displayed. In the Full-Image task, there is a great deal of clutter competing for attention and obscuring the target. By contrast, in the RSVP condition, there is far less peripheral clutter, allowing observers to focus on and more efficiently search smaller regions at a time. Given that visual clutter has been found to reduce search efficiency (Beck et al., 2010; Henderson et al., 2009; Rosenholtz et al., 2007), a reduction in clutter may result in better search performance.

Experiment 3 tested this visual clutter-based hypothesis, using stimuli similar to Experiment 2, but manipulating the level of peripheral clutter available with each RSVP segment.

## Experiment 3: the role of peripheral clutter

### Methods

#### Participants

Forty-nine participants searched for targets within both Cluttered and Uncluttered RSVP conditions.

#### Stimuli

**Fig. 4:**
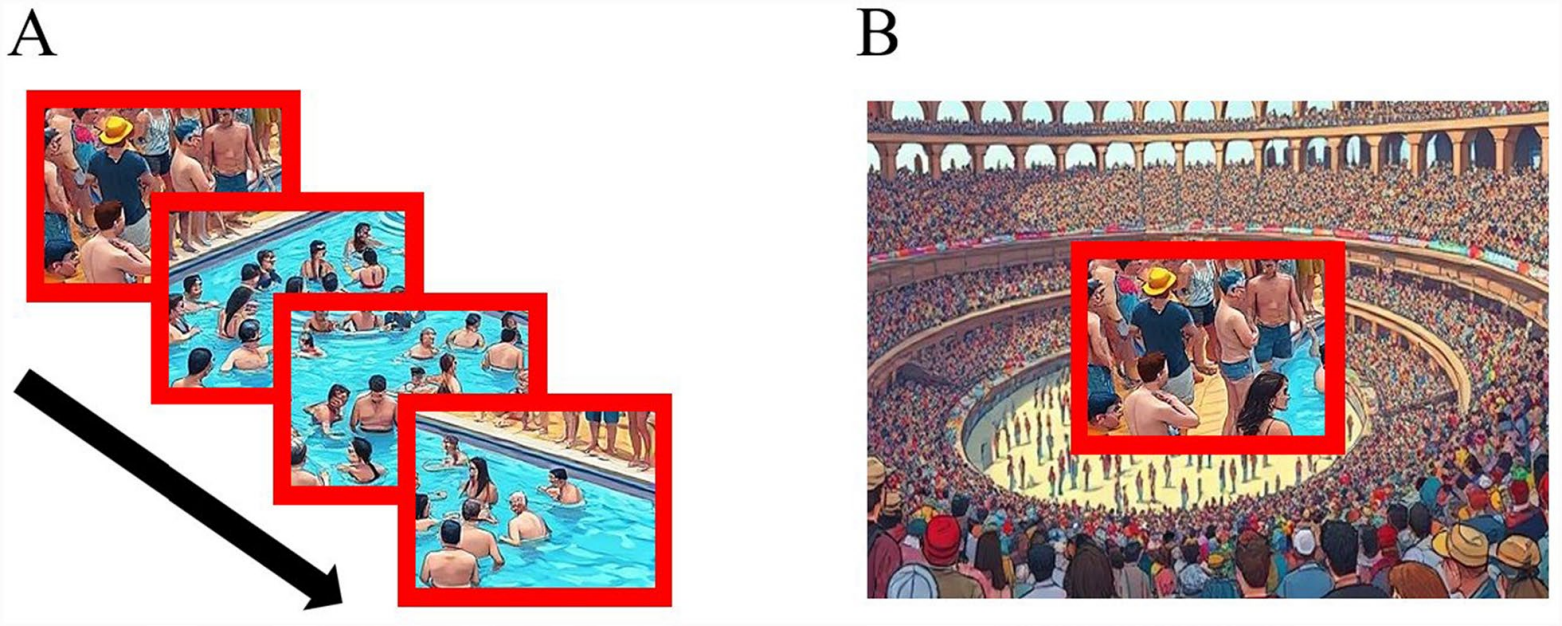
*Experiment 3 Search Stimuli.* (**A**) Uncluttered RSVP condition, in which the RSVP stream was presented at fixation, with a red outline. (**B**) Cluttered RSVP condition, in which the RSVP stream was presented amid background clutter (another Waldo image). The background clutter would remain the same throughout the duration of a trial. [Please note that due to copyright, these are AI generated replicas of the original Where’s Waldo images, used for this article only]

Stimuli were created similarly to Experiment 2, in which edited “Where’s Waldo” images were used as search scenes, and a standard “Waldo” target was present in 50% of the 16-segment RSVP scenes. Stimuli were created for two conditions: an Uncluttered RSVP and a Cluttered RSVP condition. The Uncluttered RSVP condition was identical to the RSVP condition from Experiment 2, except that each image in the RSVP stream was given a red outline of 0.5 degrees of visual angle (see Fig. 4A). The Cluttered RSVP condition was identical to the Uncluttered, except that a target-absent, whole “Where’s Waldo” image was presented behind the RSVP stream (see Fig. 4B). This peripheral image remained the same throughout the trial duration and would change upon the start of a new trial. The red outline in both conditions was implemented to denote what parts of the image were to be searched (i.e., the image/segment contained within the red outline was where the target would be located).

#### Procedure

As in Experiment 2, participants became familiar with the standard “Waldo” target and were given the goal to distinguish (via button press) when a target was present or absent. RSVP streams/trials lasted for a total of 2400 ms (150 ms per segment), after which the task prompted participants to indicate a target-present/absent response, with a new trial initiating once participants made a response. Participants completed one block of 100 trials for each condition (in which one block/condition lasted approximately 4 minutes). In all other aspects, the stimuli and procedures were the same as the RSVP condition of Experiment 2.

## Results and discussion

**Fig. 5:**
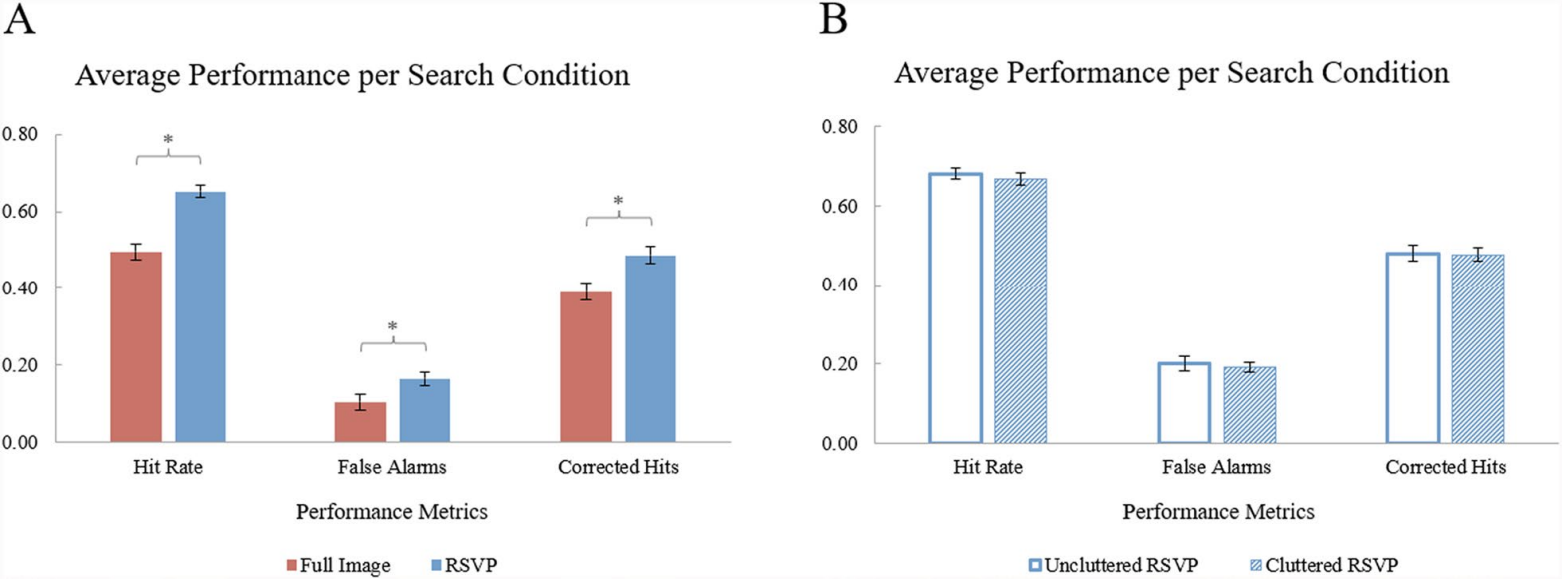
Mean hit rates, false alarm rates, and corrected hit rates (hit rate – false alarm rate) in Experiments 2 (**A**) and 3 (**B**). Errors bars represent the standard errors of the means. (*) represents a significant within-subjects comparison ($\alpha =0.05$)

No participants were excluded from analysis. There were no significant differences in performance detected between the Cluttered and Uncluttered RSVP conditions (see Fig. 5B). Paired samples t-tests failed to detect significant differences in average hit rates (*t*(48) = −1.07, *p* = .289, *d* = 0.15), false alarm rates (*t*(48) = −0.48, *p* = .631, *d* = 0.07), or corrected hit rates (*t*(48) = −0.18, *p* = .861, *d* = 0.03). These results imply that the absence of peripheral clutter was not required for an RSVP benefit.

Because no control (i.e., Full-Image) condition was investigated in Experiment 3, the following exploratory analyses—independent samples t-tests between the Full-Image and Uncluttered RSVP and the Full-Image and Cluttered RSVP conditions—were conducted to assess if RSVP advantage would persist over Full-Image search despite clutter. Thus, although the comparisons within Experiment 3 rendered null results, comparisons of performance metrics between the RSVP conditions of Experiment 3 and the Full-Image condition of Experiment 2 (see Fig. 5) revealed that RSVPs (cluttered or otherwise) continued to outperform the Full-Image condition. Experiment 3’s Uncluttered, *t*(91) = 7.58, *p* < .001, *d* = 1.58, and Cluttered, *t*(91) = 7.10, *p* < .001, *d* = 1.48, RSVP conditions yielded greater hit rates than Experiment 2’s Full-Image condition, *p*s < .001. False alarm results were similar, as Uncluttered, *t*(91) = 3.61, *p* < .001, *d* = 0.75, and Cluttered, *t*(91) = 3.78, *p* < .001, *d* = 0.79, RSVP conditions exceeded the rates of the Full-Image condition. Comparing corrected hit (hits – false alarms) rates revealed lower rates in the Full-Image than in either the Uncluttered, *t*(91) = 2.92, *p* = .004, *d* = 0.61, or Cluttered, *t*(91) = 3.10, *p* = .003, *d* = 0.64, RSVP conditions. Critically, search performance for Experiment 3’s RSVP conditions were significantly increased from the Full-Image condition, suggesting that the RSVP method improved search performance overall, despite increases in false alarms and irrespective of clutter. In short, participants could detect targets in RSVP conditions better than in a standard search, regardless of clutter.

The goal of Experiment 3 was to assess the effects of peripheral clutter on RSVP-based search performance. The extra “Waldo” backgrounds presented outside of the red outline were intended to serve as clutter for each segment and the red outlines delineated the search-relevant regions of the display. It is possible that the lack of significant differences between RSVP conditions can be attributed to the thick red outline helping participants focus their gaze to the segment contained within, minimizing the effects of peripheral clutter or distraction. This limitation of eye movements to stay within the bounds of the border may have contributed to even the Cluttered RSVP conditions surpassing Full-Image conditions in target identifications.

Additionally, in comparing RSVP performance from Experiment 3 to performance in Experiment 2, a further limitation concerns differences in peripheral visual novelty (Ernst et al., 2020) between conditions. In the Full-Image displays, each eye movement resulted in a new fixation location, altering the surrounding peripheral context and introducing novel visual information from fixation-to-fixation. In contrast, the RSVP conditions—even the Cluttered—maintained static peripheral scenes across successive segment presentations, providing a consistent visual context throughout a trial and thus less novelty to draw attention/additional fixations. This discrepancy in peripheral novelty (or the lack of novel peripheral clutter within RSVP conditions) may have influenced search behavior and target detection, potentially contributing to performance differences to full-image searches.

To more thoroughly investigate the role of eye movements during search, Experiment 4 required eye movements in the RSVP-like search condition and observed the resulting performance effects.

## Experiment 4: the role of eye movements

### Methods

#### Participants

Fifty-four participants completed visual search tasks in three conditions: a Full-Image condition and an RSVP condition which resembled the stimuli/conditions from Experiment 2, and an Uncued RSVP condition. The block order was counterbalanced.

#### Stimuli

**Fig. 6:**
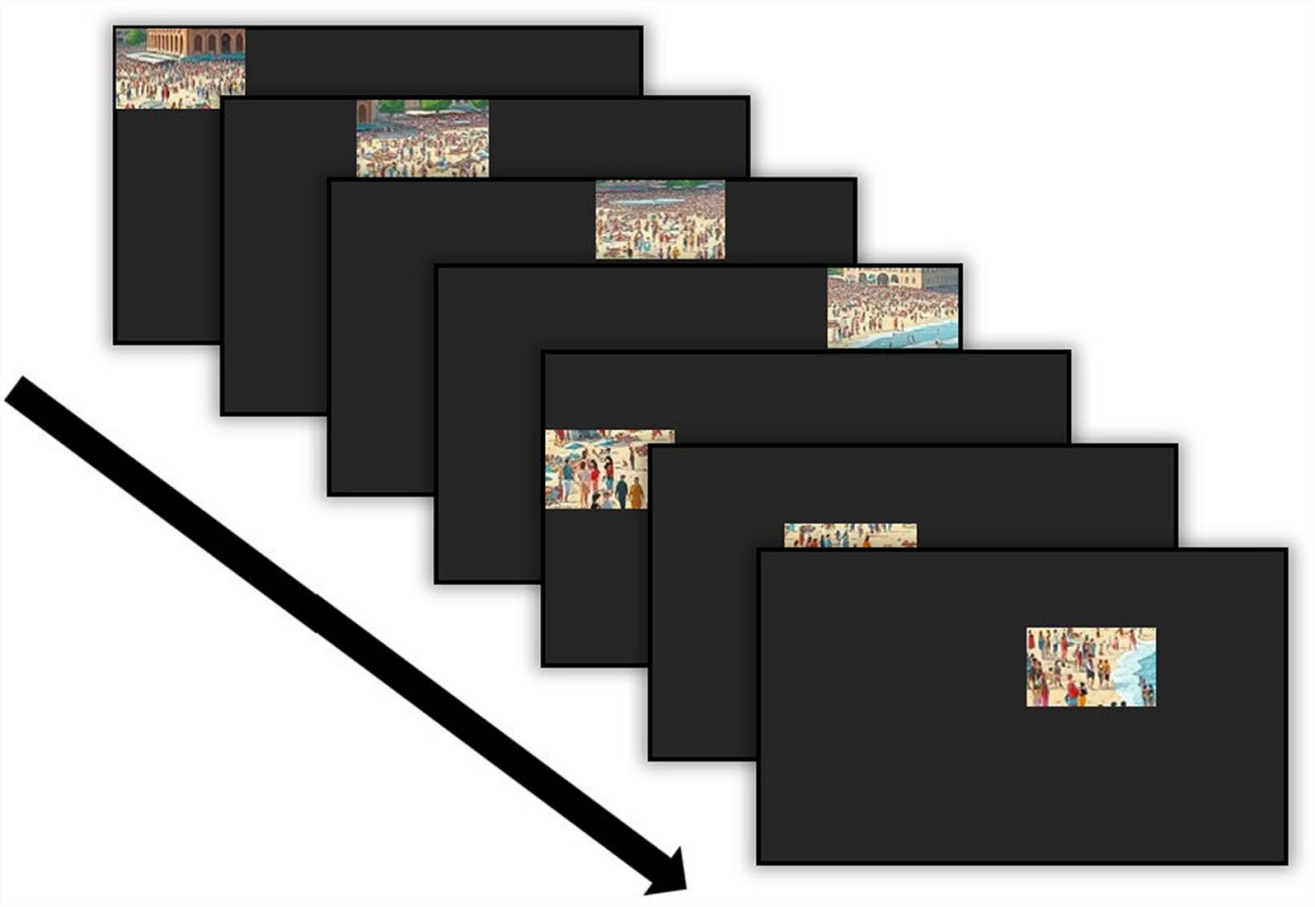
*Experiment 4 Search Stimuli. *These stimuli pertain to the Uncued RSVP condition specifically, in which a segment (1/16^th^) of a full image would be presented sequentially, from left-to-right, across the display screen. There was no external cue to direct the participant’s attention to the next image segment. [Please note that due to copyright, these are AI generated replicas of the original Where’s Waldo images, used for this article only]

The Full-Image and RSVP conditions were presented identically to Experiment 2, with the Full-Image condition consisting of 100 images/trials at 2400 ms each and the RSVP condition consisting of 16 streamed segments (each at 150 ms, at fixation) for each of the 100 stimuli images/trials. The new condition, the Uncued RSVP condition, presented segments (1/16th) of a full “Where’s Waldo” image one-at-a-time for 150 ms each (see Fig. 6). Each segment was presented one-at-a-time in a new location, with no external cue directing the participant’s attention to a new segment’s appearance (hence the label “Uncued,” in contrast to a “Cued” RSVP condition seen in Experiment 5, in which a visual cue and a time delay allowed the participant to shift their attention to the new segment’s location). The segment stream began in the top left corner of the display, proceeding to the right until reaching the end of the row, and then snaking to the row directly below it, as in a reading pattern.

The movement of the segment presentation locations provided a brief sense of segment spatial location, or position, of each segment’s content in relation to the content of other segments. This movement forced participants to 1) make additional eye movements by visually following the latest RSVP segment presentation location, as well as 2) quickly search each new segment that appeared. In each condition, targets would appear 50% of the time in a random location in the image/stream. If the need to make additional eye movements (to re-fixate the new segment) in the Uncued RSVP condition relative to the RSVP condition interfered with target identification performance, then worse overall search performance in the Uncued condition would be observed.

#### Procedure

Mirroring Experiments 2 and 3, participants performed the task of searching for the target “Waldo” in each search condition and provided a target determination response when prompted. Participants were unable to terminate trials or initiate new trials prematurely. All trials lasted for a total of 2400 ms, and participants completed 100 trials per condition in blocks that were counterbalanced.

## Results and discussion

**Fig. 7:**
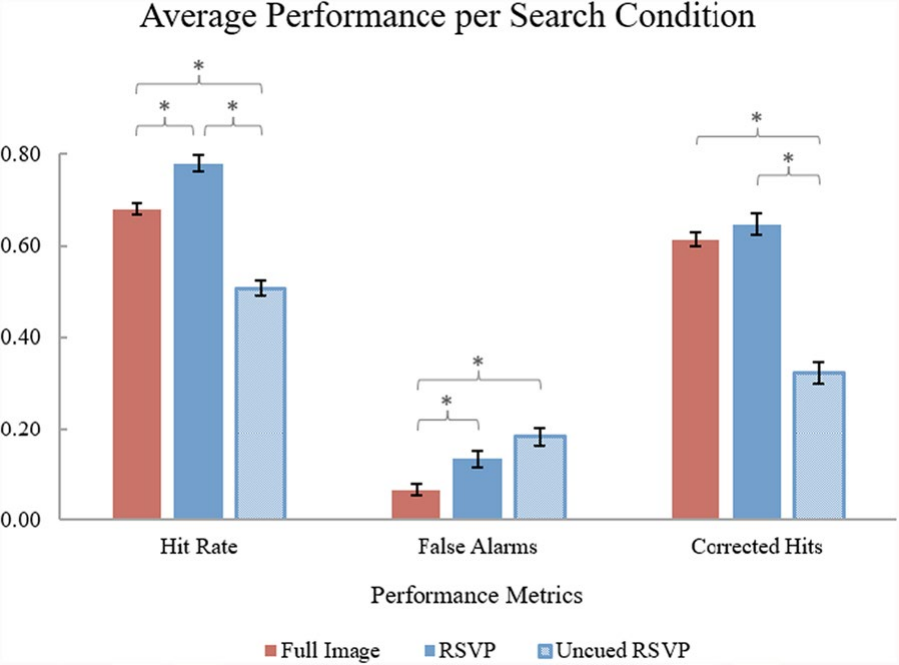
*Experiment 4 Results.* Performance metrics (hit rate, false alarm rate, and corrected hits) are compared across search conditions. Error bars represent the standard errors of the means. (*) represents a significant within-subjects comparison ($\alpha =0.05)$

Five individuals were removed from analysis for obtaining a total accuracy (hits + correct rejections) below 50%; 49 participants’ data remained for analysis. The exclusion of the five participants’ data did not change the interpretation of the overall findings. A repeated measures univariate ANOVA detected significant differences within task performance metrics. Pairwise comparisons with Bonferroni corrections helped differentiate and distinguish the main effects. Figure 7 depicts significant effects of each search condition and the performance differences between them.

Analyses detected that the RSVP (*M* = 77.96%; *SEM* = 0.02) condition had a greater hit rate, *F*(2, 96) = 154.06, *p* $<$ .001, ${\upeta }_{p}^{2}$ = 0.76, than both the Full-Image (*M* = 67.96%; *SEM* = 0.01) and Uncued RSVP (*M* = 50.69%; *SEM* = 0.02) conditions (*p*s $<$ .001). The Full-Image condition also had a significantly higher hit rate $(p < .001)$ than the Uncued RSVP condition. False alarms were significantly different across search conditions (*F*(2, 96) = 19.09, *p* $<$ .001, ${\upeta }_{p}^{2}$ = 0.28): the RSVP condition (*M* = 13.31%; *SEM* = 0.02), $p= .017$, and the Uncued RSVP condition (*M* = 18.33%; *SEM* = 0.02), $p< .001$, both had a greater number of false alarms than the Full-Image condition (*M* = 6.53%; *SEM* = 0.01). Interestingly, both the Full-Image (*M* = 61.43%; *SEM* = 0.02) and RSVP (*M* = 64.65%; *SEM* = 0.02) conditions had greater corrected hits (*F*(2, 96) = 109.33, *p* $<$ .001, ${\upeta }_{p}^{2}$ = 0.69) than the Uncued RSVP (*M* = 32.37%; *SEM* = 0.02) condition, $p\text{s} < .001$. Overall, the Uncued RSVP condition yielded the worst search performance.

Despite both conditions being limited by the need to make time-consuming eye movements, the Full-Image search yielded improved overall performance compared to the Uncued condition. It is possible that the Full-Image search provided peripheral benefit, as participants accumulated enough peripheral visual information to gauge the probability that certain areas of the search array contained a target (Wolfe, 1994; Wolfe & Van Wert, 2010). The Uncued condition had no such peripheral guidance because it did not present the search array in full (i.e., unsegmented). The Uncued condition also required time-consuming eye movements not necessary in the RSVP condition.

The additional eye movements participants made to follow and subsequently search the latest incoming segments in the Uncued condition appear to have contributed to significantly decreased target identifications relative to performance in both Full-Image and RSVP conditions. The Uncued condition’s eye movements significantly increased false alarm rates, indicating that the Uncued condition yielded the least sensitivity for target identifications (so much so that the mean hit rate for this condition was just barely above chance). This result supports the strong, negative relationship of search performance and eye movements/search efficiency in which increased eye movements are associated with poorer visual search performance (Araujo et al., 2001; Diaz et al., 2022). Additionally, this result may highlight the importance of the purpose the eye movements served during visual search. With such low overall performance, perhaps participants in the Uncued condition, after moving their eyes from the previous segment position to the latest one (i.e., making non-search-specific eye movements, in which their eyes were not actively inspecting a segment for a target) did not have enough time remaining to adequately search each segment before the appearance of the next segment.

To isolate the effects of search-specific eye movements (eye movements made within an image segment, with the goal of finding a target) versus non-search-specific eye movement (eye movements made to shift attention to a new, relevant location) in RSVP-based search, the following experiment implemented an RSVP-based search condition that still required participants to make eye movements from segment-to-segment, but preserved the search time permitted for each segment, thereby limiting the number of non-search-specific eye movements and increasing the likelihood that all segments were more thoroughly inspected.

### Experiment 5: the role of search-specific eye movements

## Methods

### Participants

Forty-four participants completed visual search tasks in four conditions in a within-subjects design. The order of conditions was counterbalanced across participants: Full-Image, RSVP, Uncued RSVP, and Cued RSVP.

### Stimuli

**Fig. 8:**
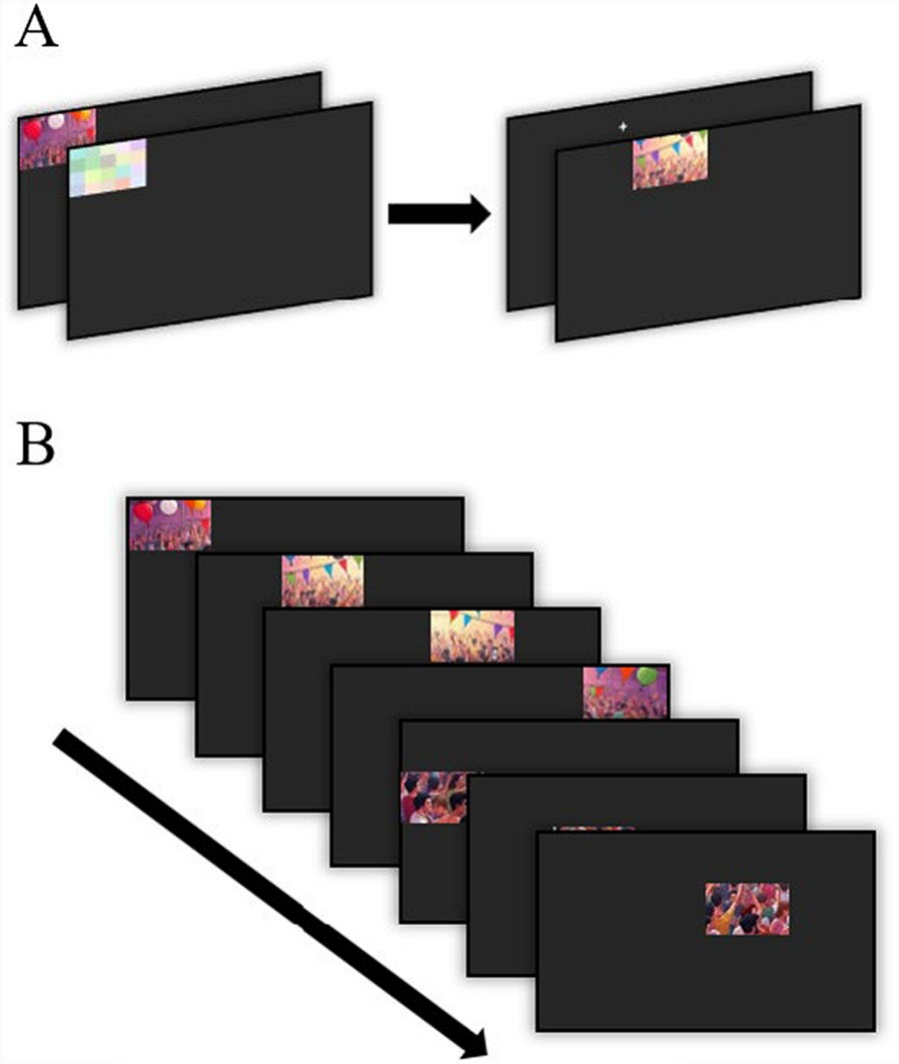
*Experiment 5 Search Stimuli. *These stimuli pertain to the Cued condition specifically, in which a segment (1/16^th^) of a full image would be presented sequentially. (**A**) The stimulus segment would be presented, followed by a mask, and in the next location a fixation cross, followed by the new stimulus segment. (**B**) Each segment/mask/fixation cross would be presented sequentially, from left-to-right, across the display screen. [Please note that due to copyright, these are AI generated replicas of the original Where’s Waldo images, used for this article only]

There were four conditions: The Full-Image and RSVP conditions were identical to those in Experiments 2 and 4, the Uncued condition was identical to that of Experiment 4, and the new condition was the Cued condition. In the Cued condition, 1/16th segments of a full “Where’s Waldo” image were presented one-at-a-time for 150 ms each. After each segment expired, a multicolored mask appeared over the segment’s location for 150 ms. The purpose of the mask was to prevent further processing of the segment area after it was removed (Liss, 1968). The mask was immediately followed by a fixation cross for 250 ms at the next segment location on the display screen (see Fig. 8A). The fixation cross allowed participants to reposition their gaze to the new segment location without expending search time. This contrasted with the Uncued condition, in which there was no time between segment presentations, and participants had to expend search time moving their eyes from the last segment location to the new one. Each Cued fixation cross/segment/mask was presented in a stream, which—like in the Uncued condition—began in the top left corner of the display and proceeded right until reaching the end of the row, in which then the next cross/segment/mask would be presented in the following row (see Fig. 8B). Participants were forced to move their eyes and follow the segment presentation locations to search each new segment that appeared.

The addition of the Cued condition tested whether the benefit of RSVP could be attributed to the reduction in task demands necessitating time-consuming eye movements. Because eye movements require periods of suppressed visual processing and consume limited search time per display, we hypothesized that performance in the Cued condition would be: (1) indistinguishable from that in the RSVP condition, in which both conditions would require minimal overt eye movements during search, and (2) greater than performance in the Uncued and Full-Image conditions, in which the spatial distribution of stimuli would require observers to frequently reorient gaze, reducing the time available for target detection. Rather than implying that eye movements inherently reduce search efficiency, our aim was to assess how minimizing their necessity might preserve limited search time within each brief display.

### Procedure

Replicating the general procedure of Experiment 4, participants completed one block of 100 trials for each search condition, and indicated whether a target was present or absent per trial when prompted.

## Results and discussion

**Fig. 9:**
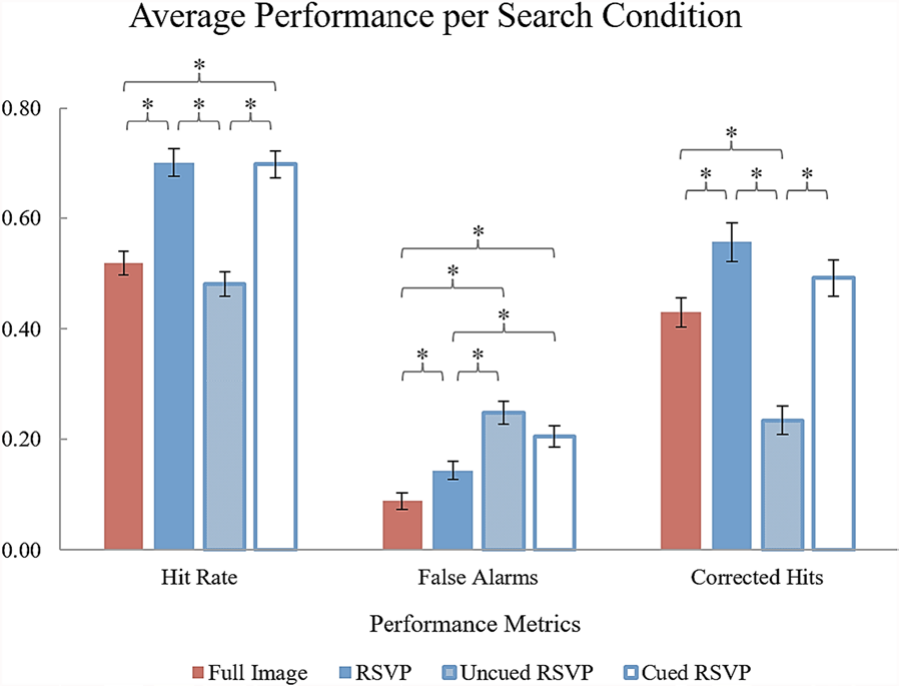
*Experiment 5 Results. *Performance metrics (hit rate, false alarm rate, and corrected hits) are compared across search conditions. Error bars represent the standard errors of the means. (*) represents a significant within-subjects comparison (α = 0.05)

Six participants’ data were excluded from analysis for total accuracy (hits + correct rejections) below 50%, leaving 38 participants’ data for analysis. The exclusion of these participants’ data did not change the overall interpretation of the data. A repeated measures univariate ANOVA detected significant differences in hit rate, *F*(3, 111) = 59.12, *p* < .001, ${\upeta }_{p}^{2}$ = 0.62, which are depicted in Fig. 9.

Pairwise comparisons with Bonferroni corrections revealed that the RSVP condition (*M* = 70.00%; *SEM* = 0.03) yielded greater hit rates on average than both the Full-Image (*M* = 51.84%; *SEM* = 0.02) and Uncued RSVP (*M* = 48.11%; *SEM* = 0.02) conditions (*p*s < .001), but was not detected to be significantly different from the Cued RSVP condition (*M* = 69.68%; *SEM* = 0.02), *p* = 1.00. The Cued RSVP condition yielded greater hit rates than the Full-Image and Uncued RSVP conditions (*p*s < .001). The Full-Image condition (*M* = 8.84%; *SEM* = 0.015) produced significantly fewer false alarms, *F*(3, 111) = 21.84, *p* < .001, ${\upeta }_{p}^{2}$ = 0.37, than the Uncued (*M* = 24.74%; *SEM* = 0.02) and Cued (*M* = 20.47%; *SEM* = 0.02) RSVP conditions (*p*s < .001). The Uncued RSVP condition had a greater false alarm rate than the RSVP condition (*M* = 14.32%; *SEM* = 0.02), *p* = .001, and the RSVP condition resulted in fewer false alarms than the Cued RSVP condition (*p* = .040). Corrected hits were significantly different between search conditions, *F*(3, 111) = 41.06, *p* < .001, ${\upeta }_{p}^{2}$ = 0.53. The RSVP condition (*M* = 55.68%; *SEM* = 0.04) yielded a greater proportion of corrected hits than the Full-Image condition (*M* = 43.00%; *SEM* = 0.03), *p* < .001, and the Uncued condition (*M* = 23.37%; *SEM* = 0.03), *p* < .001, and no observed significant difference to the Cued RSVP condition (*M* = 49.21%; *SEM* = 0.03), *p* = .351. The Uncued RSVP condition produced the worst rate of corrected hits overall, *p*s < .001.

To discern the sensitivity of each search condition to target detection performance, a univariate ANOVA, *F*(3, 111) = 15.55, *p* < .001, ${\upeta }_{p}^{2}$ = 0.30, with Bonferroni-corrected post hocs, on *d* values was calculated for each participant. This helped determine that the Full-Image (*M* = 1.24; *SEM* = 0.13), $p= .023$, RSVP (*M* = 1.68; *SEM* = 0.14), $p < .001$, and Cued RSVP (*M* = 1.49; *SEM* = 0.12), $p< .001$, conditions had increased sensitivity in comparison with the Uncued (*M* = 0.72; *SEM* = 0.09) condition.

The Cued RSVP condition’s manipulation, which may have reduced the need for time-consuming (non-search-specific) eye movements during task performance, resulted in performance that was not detected to be significantly different from the RSVP condition, and did not compromise search time across trials. Similar mechanisms between these conditions may be behind the performance benefits of these types of visual searches. A mechanism of note is eye movements, specifically efficient, search-specific eye movements. The Uncued and Cued RSVP conditions assessed if a beneficial mechanism of RSVP was the lack of non-search-specific eye movements that decreased time for visual processing of the segments. Both the Uncued and Cued conditions required participants to move their eyes to the location of a stimulus segment, but the Cued RSVP condition provided a short period of time to make eye movements without wasting search time, thereby maximizing search efficiency and the potential for the entire segment to be inspected. The Cued RSVP condition had superior hit and corrected hit rates in comparison with the Uncued RSVP condition, in which non-search-related eye movements were present and reduced total search time, resulting in performance being even worse than chance. The Cued RSVP condition’s performance was very similar to that of the RSVP condition. The evidence presented in this last experiment provides additional support for the importance of controlled, search-efficient eye movements during time constrained visual search.

## General discussion

RSVP-based search conditions tended to yield improved target identification performance (see Table 1) in each experiment’s investigation of potential mechanisms behind these performance benefits.

**Table 1:** Mean (and standard error) of performance metrics of search conditions across experiments

Experiment	Search Condition	Hits	False Alarms	Corrected Hits
1	Full-Image	62.95% (.02)	-	-
1	RSVP	69.55% (.02)	-	-
2	Full-Image	49.56% (.02)	10.49% (.02)	39.07% (.02)
2	RSVP	65.15% (.02)	16.54% (.02)	48.61% (.02)
3	Uncluttered RSVP	68.12% (.02)	20.09% (.02)	48.03% (.02)
3	Cluttered RSVP	66.82% (.02)	19.18% (.01)	47.64% (.02)
4	Full-Image	67.96% (.01)	6.53% (.01)	61.43% (.02)
4	RSVP	77.96% (.02)	13.31% (.02)	64.65% (.02)
4	Uncued RSVP	50.69% (.02)	18.33% (.02)	32.37% (.02)
5	Full-Image	51.84% (.02)	8.84% (.02)	43.00% (.03)
5	RSVP	70.00% (.03)	14.32% (.02)	55.68% (.04)
5	Uncued RSVP	48.11% (.02)	24.74% (.02)	23.37% (.03)
5	Cued RSVP	69.68% (.02)	20.47% (.02)	49.21% (.03)

Experiment 1 found that search performance was improved when each item of a search array was presented one-at-a-time in an RSVP stream relative to when the entire array was presented, even though the overall search time between the conditions was constant. This finding replicates and extends Forlines and Balakrishnan (2009), and suggests that the RSVP method of presenting stimuli can improve search accuracy. This logic was extended to more complicated search scenes in Experiment 2. Search performance for identifying “Waldo” in a “Where’s Waldo?” image was improved when the scene was cut into smaller segments and presented in an RSVP stream than when the entire image was presented. This extension of performance benefits to more complex scenes suggests that the RSVP advantage is relatively robust and can be applied to complex search tasks. Experiment 3 investigated whether the reduction in peripheral clutter could explain the RSVP advantage and found that it did not account for the increased search performance in the RSVP conditions, and that even amid peripheral clutter, RSVP offered performance benefits in contrast to searching full scenes. Experiment 4 detected a numerical trend in predicting RSVP benefit over Full-Image search, and through the introduction of an Uncued RSVP condition that required excessive eye movements that may have contributed to performance decrements, also began investigating the interaction between eye movements/search time and RSVP. Following this, Experiment 5 involved a comparison Cued RSVP eye movements condition in which eye movements were limited to search-relevant, economical movements. Performance in this condition was not distinguishable from performance on a standard RSVP, thus providing support for the role of search-efficient/search-specific eye movements involved in RSVP-based search.

RSVP performance benefits may be attributed to the RSVP method streamlining visual search by reducing or eliminating the need for extraneous eye movements during search, particularly under time constraints. For example, in the current investigation’s RSVP-based conditions, stimuli segments were presented within approximately 5 degrees of fixation, or within the fovea (Haber & Hershenson, 1973), thereby reducing the need for excessive or extended eye movements. In addition, the display duration per segment (150 ms) was faster than the duration of time that is typically required to plan and execute an eye movement (Becker & Jürgens, 1979; Greene & Rayner, 2001; Rayner, 2009). This reduction in eye movements through the use of RSVP likely increased the effective viewing time, keeping primary focus on the search array itself. In typical visual search, fixations last between 180 and 300 ms (Greene & Rayner, 2001; Rayner, 2009), and are separated by saccades which, on average, take approximately 40 ms to 50 ms (Abrams et al., 1989). Given that vision is suppressed during saccades (Rayner, 2009; Ross et al., 2001), the need to produce eye movements during search reduces the effective time to gather visual information by approximately 15% to 25%. Evidence from Experiments 1–5 also expresses an approximate 15% increase in performance when comparing RSVP-based search conditions to Full-Image conditions. The concurrence between the increase in effective search time and the increase in performance suggests the increase in performance is largely attributable to the reduction/minimization of eye movements.

The RSVP method also eliminated the ability (and need) for observers to make additional eye movements (and expend additional search time) to re-search areas of the search display. The RSVP method presents each segment of a search array once; following presentation of an RSVP stream, observers do not have to second-guess if they already searched a segment/area of the array. This streamlining enhances the efficiency of visual search and decreases the memory load inflicted onto observers (when compared to Full-Image searches). Overall, the RSVP benefit may be driven by the reduced need for eye movements, thereby maximizing the amount of time during a trial in which information is being analyzed from the visual array. To verify these inferences, future studies can employ eye tracking and monitor ocular effects of RSVP-based visual search.

Although it was found that the RSVP technique significantly improved target detection, Experiments 2–5 revealed that RSVP-based conditions increased false alarms, which may have negative implications for the real-world application of this RSVP technique. In radiology, baggage, and military screenings, targets are rare (Gur et al., 2004), meaning that even a small increase in false alarm rates could result in a large increase in the absolute number of false alarms. Although an increase in false alarms may be problematic, the corrected hits of the RSVP-based conditions in the current study did account for the overall performance tradeoff in increased hits vs. increased false alarms, i.e., RSVP-based conditions still significantly outperformed the Full-Image conditions. The tradeoffs never leaned heavily toward increased false alarms at the expense of correct target identifications, only indicating increased sensitivity. Moreover, the fact that increased false alarms represent a liberal shift in criterion may actually be a benefit of the RSVP technique: increased applicability toward mitigating different causes of performance decrements—although this benefit would be task dependent.

For example, the extent of RSVP benefits may change depending on the prevalence of visual search targets. When performing low target prevalence search (i.e., search in which targets are rare), observers tend to use a more conservative decision criterion (Wolfe & Van Wert, 2010; Wolfe et al., 2007). The current study, which employed a target prevalence of 50% across experiments, suggests that RSVP may prompt more liberal decision criterion, which may mitigate the performance effects of low target prevalence. In high-prevalence search, observers may demonstrate liberal decision criterion, resulting in increased false alarms (Menneer et al., 2010; Wolfe & Van Wert, 2010)—RSVP’s shift toward liberal criterion may exacerbate high-prevalence effects. Therefore, the benefits of RSVP may vary, depending on target prevalence. Many real-world tasks involve searching for rare targets (e.g., searching for a foreign vessel on a sonar display), and the utility/effectiveness of RSVP in these types of tasks has yet to be seen. Future experimentation should assess bias, accuracy, and sensitivity measures in a low-prevalence (Wolfe et al., 2007) RSVP search to reflect conditions of real-world search scenarios.

Real-world visual search may also involve prolonged or cognitively fatiguing searches (e.g., sonar monitoring), in which RSVP performance effects may differ. Despite the RSVP method presenting segments near fixation and within the focal field of vision, observers must sustain conscious attention to search the fixated segment and determine the presence of any target(s) (Peltier & Becker, 2016). Such attention may diminish amid additional cognitively fatiguing factors found in operationally relevant search scenarios, including sleep deprivation (De Gennaro et al., 2001) or time-on-task fatigue (Mackworth, 1948). The current study implemented conditions in which total search times were four to five minutes in duration; such durations should be extended in future studies to investigate the effects of cognitive fatigue on target identification performance during the implementation of different stimulus presentation methods (i.e., RSVP vs. Full-Image presentations).

Additionally, future research should explore how the observed advantages of RSVP vary across different presentation durations and temporal parameters. The current study used a fixed RSVP presentation duration (150 ms per segment, 2400 ms total) and did not investigate whether this duration reflects the optimal or maximal difference in visual search performance between RSVP and Full-Image displays. One promising approach could involve implementing an adaptive timing procedure in which search duration automatically adjusts to maintain a target level of observer accuracy—such as 75%. Following a correct response, the duration would be reduced, whereas incorrect responses would trigger an increase in search time. This method would allow researchers to identify the lower and upper bounds of effective RSVP presentation times and how those thresholds vary with variables including task difficulty, total task duration/total number of trial blocks, etc. Moreover, such a performance-driven design would provide insight into RSVP speed-accuracy tradeoff, in how much faster (rather than merely how much more accurate, as in the current study) RSVP can be relative to Full-Image search, offering a more nuanced understanding of its potential benefits in applied, high-stakes search environments.

Such high-stakes environments (e.g., battlefield surveillance via drones) often require a certain level of observer expertise and experience that guides visual search (Godwin et al., 2015a; Guznov et al., 2017). It is worth noting, though, that the stimuli/arrays used in the current study were extremely difficult searches that offered little opportunity for search guidance, and therefore the utility of RSVP in such guided search or context-specific search may be limited. According to Wolfe’s guided search model (Wolfe, 1994, 2021), visual search is a two-stage process. The initial parallel stage involves the identification of locations that share properties with the target and are thus likely target locations. The second stage involves the serial deployment of attention to those items (or groups of items)/areas. In the visually cluttered Full-Image arrays used across experiments presented in the current study, the density and complexity of visual information may have overwhelmed the initial parallel stage, rendering it largely ineffective. Target-similar characteristics likely were not located or were not all processed in time due to the sheer volume of objects in the stimulus images/arrays. These environments created favorable conditions for demonstrating the advantages of RSVP and arrays for which the performance benefits of a scene-wide, initial parallel stage of guided search are limited.

However, this experimental design may limit the generalizability of the current study’s findings to real-world visual search tasks and environments in which target locations may not be randomly distributed and are often constrained by the semantic or structural context of the scene. In naturalistic settings, contextual information plays a critical role in guiding attention to plausible target locations. For example, in everyday environments, people do not search for a cup on the ceiling or for pedestrians in the sky. In higher-stakes operational search environments, expert radiologists, for example, use peripheral vision, landmarks, and prior knowledge to guide their attention/search (Nodine et al., 1996). Prior knowledge and spatial regularities help filter out unlikely locations and prioritize relevant ones.

A similar “filter-out” phenomenon may have been present in Experiment 3, which investigated the role of peripheral visual clutter in RSVP benefit. Although full-image backgrounds accompanied the segments in the Cluttered RSVP condition to approximate the visual complexity of a full-image search, these background images never contained a target and were therefore not task-relevant. In contrast, in the Full-Image conditions, peripheral regions could have contained the target and may have attracted attention as participants planned their eye movements. As such, the backgrounds in the Cluttered condition may not have engaged attention in the same way, prompting participants to filter out the “clutter” and prioritize the segments, potentially limiting the strength of the comparison. Future work could address this by embedding task-relevant information throughout the periphery of the scene, even in Cluttered condition format, to more closely parallel the demands of a full-image search.

By reducing the peripheral guidance that may be available in the overall search array, it is possible that an RSVP method would eliminate not only visual cues, but also spatial cues that observers need to perform their jobs/searches effectively. Though Experiment 4 and 5’s Uncued/Cued RSVP conditions did attempt to provide some semblance of spatial relationships between segments and the search objects that comprised them, this information was not evidenced to provide search benefit beyond standard RSVP, and was likely difficult to retain amid making eye movements to keep up with and subsequently search the segment presentations. Spatial and contextual guidance enables more efficient search and may be particularly important when targets are small, infrequent, or embedded in noisy scenes.

A full-image search method might therefore be more efficient than an RSVP search in cases where the initial parallel stage or peripheral guidance is effective in guiding search (e.g., with bodily scans that provide visual evidence/symptoms pointing toward the presence of an abnormality). The benefit of an RSVP task is likely to depend on the search task. For example, in a task that requires search for a particular color target, in which guidance is highly effective at constraining search to stimulus items that match that target color (Hamblin-Frohman et al., 2023), an RSVP technique may be less helpful than a full-image search. The utility and benefit of RSVP therefore should be researched in greater contexts. 

## Conclusions

The results of the current effort demonstrate that the RSVP presentation method can improve target detection in searches of both simple arrays and complex scenes. This improvement could be attributed to a reduction in the need to perform extraneous eye movements in the RSVP condition. These findings also suggest that this technique may be a promising method for improving target detection in real-world search scenarios, depending on the task. The promising RSVP technique should be tested to determine if it yields similar benefits in cases where target prevalence is reduced, search guidance is more effective, etc. These five studies imply that RSVP may be a realistic method to improve search, but further research with experienced observers and more realistic, operationally relevant stimuli is necessary to fully understand the applicability of and mechanisms behind RSVP-based search. Upon verification of these various mechanisms of improving visual search, they can be implemented into real-world visual search tasks and systems, enhancing target identification across a wide span of duties.

## Data Availability

The data generated from and analyzed during the experiments are available from the corresponding author upon reasonable request.

## Abbreviations

ANOVA: Analysis of variance
M: Mean
MSU: Michigan State University
MRI: Magnetic resonance imaging
ms: Milliseconds
RSVP: Rapid serial visual presentation
SEM: Standard error of the mean
TSA: Transportation security administration
UUV: Unmanned underwater vehicle
